# Italian Guidelines for Cardiological Evaluation in Competitive Football Players: A Detailed Review of COCIS Protocols

**DOI:** 10.3390/healthcare13151932

**Published:** 2025-08-07

**Authors:** Umile Giuseppe Longo, Georg Ahlbaumer, Roberto Vannicelli, Emanuele Gregorace, Davide Ortolina, Guido Nicodemi, Daniele Altieri, Arianna Carnevale, Silvia Carucci, Alessandra Colella, Francesco Scalfaro, Erika Lemme

**Affiliations:** 1 Fondazione Policlinico Universitario Campus Bio-Medico, Via Alvaro del Portillo, 200, 00128 Roma, Italy; g.longo@policlinicocampus.it (U.G.L.); biomex@compunet.ch (G.A.); roberto.vannicelli@asroma.it (R.V.); emanuele.gregorace@asroma.it (E.G.); davide.ortolina@asroma.it (D.O.); nicodemiguido@gmail.com (G.N.); daniele.altieri@unicampus.it (D.A.); s.carucci@policlinicocampus.it (S.C.); a.colella@policlinicocampus.it (A.C.); f.scalfaro@policlinicocampus.it (F.S.); e.lemme@policlinicocampus.it (E.L.); 2 Research Unit of Orthopaedic and Trauma Surgery, Department of Medicine and Surgery, Università Campus Bio-Medico di Roma, Via Alvaro del Portillo, 21, 00128 Roma, Italy; 3 A.S.Roma, Piazzale Dino Viola, 1, 00128 Roma, Italy; 4 Klinik Gut St. Moritz, Plazza Paracelsus 2a, 7500 St. Moritz, Switzerland

**Keywords:** sudden cardiac death, hypertrophic cardiomyopathy, electrocardiogram

## Abstract

**Background:** Medical clearance for competitive sports is vital to safeguarding athletes’ health, particularly in high-intensity disciplines like football. In Italy, fitness assessments follow stringent protocols set by the Commissione di Vigilanza per il controllo dell’Idoneità Sportiva (COCIS), with a strong focus on cardiovascular screening. The primary goal is to prevent sudden cardiac death (SCD), a rare but catastrophic event in athletes. **Methods:** This paper provides an in-depth narrative review of the 2023 COCIS guidelines, examining the cardiological screening process, required diagnostic tests, management of identified cardiovascular conditions, and the protocols’ role in reducing SCD risk. **Results:** Comparisons with international standards underscore the effectiveness of the Italian approach. **Conclusions:** The COCIS 2023 guidelines provide clear, evidence-based protocols for cardiovascular risk assessment, significantly enhancing athlete safety and reducing the incidence of SCD in high-intensity sports.

## 1. Introduction

Medical certifications are a crucial component of athlete safety, especially in competitive sports that demand high physical exertion [1,2]. Football, characterized by intense physical stress, places significant strain on the cardiovascular system, making it essential to identify athletes at risk of SCD [3]. Italy’s mandatory certification process exemplifies a proactive approach, establishing the country as a potential benchmark in sports medicine and cardiology. In contrast, international standards may not always mandate such rigorous evaluations [4].

The history of sports-related SCD has profoundly influenced the development of these guidelines. Since the introduction of mandatory screening in the 1980s, Italy has achieved a marked reduction in the incidence of SCD among young athletes. This success has attracted international recognition, serving as a model for other countries seeking effective preventive strategies. The decline in SCD rates underscores not only the effectiveness of the protocols but also the critical importance of consistent follow-up and monitoring—particularly for young athletes who may be unaware of underlying cardiovascular conditions [5].

This accomplishment largely stems from early detection of cardiovascular anomalies through routine physical examinations, resting ECGs, and stress test ECGs, all of which are now mandatory components of fitness certification for competitive athletes. The COCIS protocols have been periodically updated to incorporate advances in medical science; the most recent 2023 revision introduced enhancements that emphasize early diagnosis and preventive care for at-risk athletes, along with more stringent guidelines regarding the utilization of advanced diagnostic tools and the management of arrhythmias and cardiomyopathies. These updates include detailed risk stratification, enabling physicians to tailor recommendations to each athlete’s specific health profile, thereby ensuring a more precise and individualized certification process.

Furthermore, the COCIS protocols stand out as one of the few contemporary frameworks, offering personalized evaluations of permissible physical activity for patients with known cardiovascular conditions, such as arterial hypertension, valvular disease, and arrhythmias.

## 2. Sudden Cardiac Death in Athletes: Understanding the Risk

Sudden Cardiac Death (SCD) is the leading cause of mortality in young athletes. It occurs at a rate of approximately 1 in 50,000 athletes per year [6]. Football is considered a high-risk sport. This is due to its combination of intense aerobic and anaerobic demands, rapid directional changes, and sudden accelerations. The sport requires explosive sprints, abrupt stops, and physical contact. These actions place significant strain on the heart, especially in athletes with underlying cardiac conditions [7]. Understanding how the demands of football interact with cardiac physiology is essential for developing effective prevention programs. Most cases of SCD are caused by previously undiagnosed cardiovascular conditions. These conditions are often asymptomatic, which makes early detection critical [5]. Routine screenings and diagnostic evaluations can help identify these conditions before they result in fatal events [8]. However, many of these disorders—such as hypertrophic cardiomyopathy (HCM) and ion channelopathies—can go undetected during standard health screenings. This highlights the need for targeted, sport-specific cardiovascular assessments.

Primary Causes of SCD in Athletes (Table 1):

- Hypertrophic Cardiomyopathy (HCM): HCM is a genetic disorder characterized by thickening of the left ventricular wall. This condition increases the risk of arrhythmias and sudden cardiac arrest [9]. It is the most common cause of SCD in young athletes, particularly during physical exertion. HCM is especially prevalent in Afro-Caribbean athletes. This underlines the importance of early and regular screenings. The hypertrophy seen in HCM may resemble normal adaptations in elite athletes. Therefore, distinguishing between physiological adaptation and pathological hypertrophy is a key focus of the COCIS protocols. Mild cases of HCM may have no noticeable symptoms. As a result, comprehensive imaging is often required for diagnosis [10]. Regular monitoring helps ensure that even athletes with subtle indicators receive proper evaluation.
- Arrhythmogenic Right Ventricular Cardiomyopathy (ARVC): ARVC is a genetic condition that gradually replaces heart muscle with fibrous or fatty tissue. It typically affects the right ventricle but can also involve the left or both ventricles. This condition increases the risk of arrhythmias and SCD, especially in individuals between the second and fourth decades of life [11]. In Europe, the prevalence of ARVC is estimated to range from 1 in 5000 to 1 in 2000. ARVC may remain unnoticed until symptoms occur during intense exercise. Common symptoms include palpitations, syncope, and chest pain. The Italian protocol recommends advanced imaging if any major ECG abnormalities are observed. This is especially important when abnormalities are combined with family history, stress test irregularities, or arrhythmias. Ventricular dysfunction often only becomes evident under exercise-induced stress. Cardiac MRI is particularly useful for detecting early signs of ARVC, as electrical anomalies may only appear under high-stress conditions [12]. Given its often silent progression, athletes with even minor ECG changes should receive routine follow-up evaluations.
- Congenital Coronary Artery Anomalies: These are structural abnormalities in the coronary arteries present from birth. They can reduce blood flow to the heart muscle during exertion, leading to ischemia and fatal arrhythmias [13]. Unlike acquired coronary artery disease, congenital anomalies often require advanced imaging to detect. CT coronary angiography is increasingly recommended in cases where traditional screening methods are inconclusive. The COCIS protocols support the use of coronary CT angiography when congenital abnormalities are suspected, as they may not be visible on standard imaging.
- Ion Channelopathies (e.g., Long QT Syndrome, Brugada Syndrome): Ion channelopathies are inherited disorders that affect the heart’s electrical activity. They increase the risk of life-threatening arrhythmias, particularly during intense physical activity [14]. High-intensity exercise alters the electrical and autonomic balance of the heart, which can trigger symptoms in athletes with underlying channelopathies. Diagnosis requires a multidisciplinary approach. This includes clinical assessment, resting ECG, exercise testing, 24 h Holter monitoring, pharmacological stress tests, and genetic analysis. Genetic testing is now a core part of the COCIS protocols. Early identification allows for risk mitigation through personalized training plans, close monitoring during competition, or pharmacological therapy when necessary.

Rare Causes of SCD in Athletes (Table 2):

- Andersen–Tawil Syndrome (LQTS Type 7): Andersen–Tawil Syndrome is a rare genetic disorder caused by mutations in the KCNJ2 gene. It typically presents with mild to moderate QT interval prolongation. U waves are frequently observed. Characteristic arrhythmias include bidirectional ventricular tachycardia. The risk of sudden cardiac death exists but is generally considered low. Prevalence is approximately 1 in 1,000,000.
- Timothy Syndrome (LQTS Type 8): Timothy Syndrome is a severe, ultra-rare condition caused by mutations in the CACNA1C gene. It features extreme QT prolongation (often > 600 ms), frequent AV block, and high risk of malignant arrhythmias. Patients show multisystem involvement. Sudden cardiac death risk is extremely high, often occurring in early childhood. Prevalence is around 1–2 per 100 million individuals.

## 3. The Role of COCIS Protocols in Preventing SCD

The COCIS protocols are designed to detect underlying cardiovascular conditions that may predispose athletes to sudden cardiac death. The primary aim is to identify asymptomatic, at-risk athletes through a systematic and mandatory screening process in order to detect underlying conditions—that might otherwise go unnoticed—and prevent potential fatalities. These protocols have evolved over time and now consist of multiple phases of evaluation, including medical history, physical examination, ECG screening, and additional diagnostic tests for those with abnormal findings. This structured approach has proven effective: in the period from 1979–1980 to 2003–2004, the annual incidence of sudden cardiovascular death in athletes decreased by 89% (from 3.6/100,000 person-years in 1979–1980 to 0.4/100,000 person-years in 2003–2004; *p* for trend < 0.001) [15].

Each phase of this approach is designed to progressively narrow down potential risk factors, leading to a more precise and comprehensive assessment. Additionally, COCIS emphasizes a holistic approach to athlete care, incorporating family history, lifestyle factors, and detailed cardiac monitoring.

The protocols are particularly focused on identifying key risk factors for SCD, such as HCM, coronary artery anomalies, and electrical disorders like long QT syndrome. For athletes involved in high-risk sports like football, the COCIS protocols mandate further investigations, including echocardiograms and Holter monitoring, to assess the full extent of any detected abnormalities. Furthermore, recent advancements in imaging techniques, such as the integration of late gadolinium enhancement (LGE) in MRI protocols, have provided clinicians with enhanced diagnostic clarity in assessing myocardial scarring or fibrosis, markers often associated with increased arrhythmic and SCD risk.

The implementation of these protocols has been shown to significantly reduce the incidence of SCD in Italian athletes, highlighting the efficacy of COCIS protocols as a model for other nations, which involve less rigorous screening protocols: for instance, the AHA/ACC guidelines prescribe a medical history and physical examination, with an ECG conducted only when clinically indicated by the physician. The Italian experience illustrates the value of adopting a national, continuous, standardized approach, providing compelling evidence that mandatory cardiovascular screening can have a profound impact on athlete health and safety, with broader implications for public health.

## 4. Cardiovascular Screening Protocols in Competitive Football

Football, as a sport that involves significant cardiovascular stress, places athletes at a higher risk for SCD [16,17]. The COCIS protocols provide a step-by-step screening process designed to capture both electrical and structural abnormalities in the heart. The screening process involves a combination of medical history reviews, resting ECGs, stress tests, and advanced diagnostic tools such as echocardiography and cardiac MRI. Each step is crucial in identifying potential risks and determining whether an athlete is fit to compete. Given the unique stressors football places on the cardiovascular system, COCIS protocols include tailored recommendations to assess both short- and long-term adaptations of the athlete’s heart, ensuring a sport-specific deep evaluation.

### 4.1. Medical History and Initial Cardiovascular Evaluation

The first step in the cardiovascular evaluation process is a thorough review of the athlete’s medical history. This review focuses on identifying any personal or familial indicators of cardiovascular disease, including:

- Family history of sudden cardiac death, particularly in relatives under 50 years of age.
- Personal history of syncope, especially if it occurs during physical activity.
- Symptoms such as palpitations, chest pain, or abnormal shortness of breath during exercise.
- Prior diagnoses of heart conditions such as high blood pressure, arrhythmias, or congenital heart disease.

By thoroughly reviewing these indicators, physicians can identify athletes who may be at higher risk for conditions like HCM or ARVC, even before conducting any tests. The COCIS protocols suggest that physicians assess any history of unexplained fainting episodes, as such incidents often correlate with underlying cardiac abnormalities, requiring further examination to rule out electrical or structural issues. For young athletes, a detailed family history is particularly valuable, as certain inherited cardiac conditions may remain undetected without symptoms until the athlete is under high physical demand. The COCIS protocols also suggest re-evaluating medical histories periodically to identify new developments that could influence cardiac risk.

### 4.2. Resting ECG

A 12-lead resting ECG is the cornerstone of the COCIS protocols. This non-invasive test provides essential information about the electrical activity of the heart, detecting potential arrhythmias or signs of hypertrophy that may predispose an athlete to SCD. The high sensitivity of ECG in identifying early markers of HCM and long QT syndrome allows for timely intervention before the onset of severe symptoms. The protocol mandates that even mild ECG abnormalities trigger further diagnostic steps, ensuring no potential risks are overlooked. Additionally, the ECG results serve as a baseline for future comparisons, particularly valuable in tracking cardiac adaptations that could signify underlying pathologies in response to intense physical training.

ECG screening has been shown to be highly effective in detecting asymptomatic conditions such as hypertrophic cardiomyopathy and long QT syndrome [18]. Athletes with abnormal findings are typically referred for further diagnostic evaluation, including stress tests or echocardiograms. The Italian COCIS protocols have refined criteria to differentiate physiological changes, such as increased left ventricular mass in highly trained athletes, from pathological changes that require follow-up. This differentiation minimizes unnecessary disqualification and anxiety while maintaining stringent safety standards.

The routine use of ECG in Italy has led to a significant reduction in SCD among athletes. A 25-year study of Italian athletes demonstrated a decline in the incidence of SCD, with many athletes being diagnosed with potentially fatal conditions through routine ECGs. Italian protocols have integrated updates based on research, which recommends a minimum of yearly ECGs for active competitive athletes, particularly in high-intensity sports such as football.

### 4.3. Exercise Stress Test

This test assesses how the heart responds to increased physical activity, monitoring for signs of ischemia, arrhythmias, or abnormal blood pressure responses. Exercise stress testing under the COCIS protocol provides an invaluable tool to simulate game-like stressors on the cardiovascular system, helping to identify hidden vulnerabilities that may not be evident at rest. The exercise stress test is routinely performed as part of the cardiovascular screening process. For athletes who show signs of cardiovascular abnormalities on their resting ECG or report symptoms like chest pain during physical exertion, the exercise stress test assumes even more importance as the next step.

During the test, athletes are required to perform gradually increasing physical exercise on a treadmill or stationary bike while their heart rate, ECG, and blood pressure are continuously monitored. Abnormalities detected during the test may prompt further investigation with advanced diagnostic imaging such as echocardiography or cardiac MRI. The stress test protocol in Italy includes measurements of immediate recovery heart rate, an indicator that has been associated with heightened cardiovascular risk when abnormally prolonged.

### 4.4. Echocardiogram

An echocardiogram is a non-invasive imaging test that uses ultrasound to create detailed images of the heart’s structure and function [14]. It is particularly valuable for detecting structural abnormalities such as HCM or dilated cardiomyopathy (DCM), both of which can increase an athlete’s risk of SCD. COCIS protocols recommend that echocardiograms be used to examine left ventricular wall thickness, chamber size, and cardiac output, as these measurements are part of a differential diagnosis, distinguishing athletic heart adaptations from pathological changes.

In athletes showing abnormal ECG results, echocardiography is often used to confirm the diagnosis and assess the severity of any detected abnormalities. For example, in cases of suspected HCM, the echocardiogram may reveal thickening of the heart walls, particularly the left ventricle. Additionally, Italian protocols suggest repeat echocardiography in cases where borderline structural changes are observed, enabling longitudinal monitoring of cardiac adaptations and early intervention if pathological changes progress. This approach ensures that cardiac conditions like HCM are diagnosed at an early, manageable stage, ultimately reducing the risk of SCD.

### 4.5. Holter Monitoring and Cardiac MRI

Holter monitoring is used to record the electrical activity of the heart over a 24–48 h period [19]. It is particularly useful in detecting intermittent arrhythmias that may not appear during a standard resting ECG. Athletes who experience sporadic palpitations, dizziness, or syncope during physical activity may benefit from this extended monitoring, which can provide insights into arrhythmias triggered by exertion. Holter monitoring offers detailed, time-based insights into heart rhythm variability over extended periods, making it a valuable tool in identifying patterns of arrhythmia that might only manifest during specific exertional phases or recovery periods.

Cardiac MRI is used for detailed imaging of the heart, particularly in cases where echocardiography has raised concerns about structural abnormalities [20]. MRI can detect scar tissue, fibrosis, and subtle signs of ARVC. This imaging modality is also used to assess heart muscle inflammation in conditions like myocarditis, which can increase the risk of SCD in athletes.

## 5. Literature Review and International Comparison

Worldwide, the use of ECG screening is still not standardized. In the United States, pre-participation evaluations rely primarily on medical history and physical examination, without the mandatory inclusion of ECG. In Norway, cardiovascular screening is not mandatory for all athletes; instead, a targeted screening approach is adopted for high-risk groups. While other international guidelines, such as those from the ESC and AHA, emphasize individualized medicine—often leaving the final decision to clinical judgment and the athlete’s informed preference—the COCIS protocol is more prescriptive, prioritizing legal protection and consistency in eligibility determinations (Table 3). Critics of the Italian approach argue that the high rate of false positives can lead to unnecessary testing and anxiety [21], although recent improvements in ECG interpretation criteria, such as the International Criteria, have significantly reduced this percentage. Moreover, supporters of ECG screening contend that the ability to detect potentially fatal conditions justifies the approach. In the United Kingdom, ECG screening is increasingly used in high-risk sports; however, the implementation is not as uniform or extensive as in Italy. The ongoing debate over cost-effectiveness versus the potential to save lives continues to influence international sports medicine practices. Countries adopting similar strategies report decreased mortality: a meta-analysis conducted on data from 28,011 athletes screened between 2015 and 2020 demonstrated that the odds of detecting cardiac disease through pre-participation ECG are statistically significant, with an odds ratio of 60 (*p* < 0.001); in contrast, the odds of detection through history and physical examination alone were markedly lower and not statistically significant, with an odds ratio of 3.4 (*p* = 0.076) [22]. These findings further underscore the cost-effectiveness of incorporating ECG into preparticipation screening protocols. Specifically, the addition of ECG is estimated to save 2.06 life-years per 1000 athletes, with an incremental cost of only $89 per athlete [23], rendering it a financially and clinically justifiable strategy when compared to the substantial costs associated with managing cardiac emergencies. Italian findings underscore the life-saving potential of mandatory cardiovascular assessments and serve as a model for sports medicine policies worldwide.

## 6. Discussion

The implementation of the COCIS protocols could prompt considerations regarding their practicality, cost-effectiveness, and psychological impact on athletes. One concern revolves around the financial implications of mandatory comprehensive screenings, particularly the routine use of ECGs and advanced diagnostic tests. The expenses associated with widespread ECG screening, especially in countries with large athlete populations or constrained healthcare budgets, may be prohibitive [24]. The costs of additional testing resulting from false-positive results could strain resources and may not be justifiable given the relatively low incidence of SCD.

However, extensive research supports the cost-effectiveness of such screenings in the long term. A study published in 2010 by Wheeler et al. [23] demonstrated that pre-participation cardiovascular screening with ECG is cost-effective when considering the quality-adjusted life years saved through the prevention of SCD. Furthermore, the societal and emotional costs of losing young athletes to preventable cardiac events far exceed the monetary investments in preventive measures. The Italian experience remarks on this point, as the reduction in SCD incidence among athletes not only saves lives but also reduces long-term healthcare costs associated with managing cardiac events and their aftermath [15].

Another concern is the potential psychological impact on athletes who are identified as having cardiac abnormalities. The detection of such conditions can lead to anxiety, stigmatization, and, in some cases, the premature end of athletic careers [21]. This is particularly challenging for young athletes whose identities and future aspirations are closely tied to sports participation. By providing clear communication about the nature of the findings, the implications for the athlete’s health, and potential management strategies, healthcare professionals can help athletes make informed decisions while minimizing undue stress [2].

The issue of false positives in ECG screening is also a point of contention [25,26]. Early studies reported high false-positive rates, leading to unnecessary additional testing and temporary disqualification from sports. However, advancements in ECG interpretation criteria specifically tailored for athletes have significantly improved the specificity of screenings. The development of the “Seattle Criteria” [27] and the “International Recommendations for ECG Interpretation in Athletes” [28] have reduced false-positive rates by distinguishing between physiological adaptations to exercise and pathological findings. In 2019, in a cohort of 5258 NCAA athletes, the total number of ECGs flagged as abnormal by expert over-read decreased from 158 (3.0%) using the Seattle Criteria to 83 (1.6%) using the International Criteria (*p* < 0.0001) [29]. Incorporating these refined criteria into the COCIS protocols enhances their accuracy and efficiency, ensuring that athletes are not unfairly excluded from participation [30].

The adaptability of the Italian model to other countries with different healthcare infrastructures is another consideration [31,32]. Italy’s centralized healthcare system facilitates uniform implementation of the COCIS protocols nationwide. In contrast, countries with decentralized systems or varying levels of access to healthcare resources may face challenges in adopting similar measures. Despite these challenges, the core principles of the COCIS protocols—early detection, comprehensive evaluation, and personalized risk assessment—can be adapted to different contexts. Collaborative efforts, such as international consensus statements and shared best practices, can aid in tailoring the protocols to suit various healthcare settings while maintaining their effectiveness. Some strategies could facilitate adaptation (e.g., pilot programs, phased implementation).

Moreover, technological advancements offer promising avenues to enhance the feasibility and reach of cardiovascular screenings [28,33]. Portable ECG devices and telemedicine platforms can extend access to remote or underserved areas, reducing disparities in athlete healthcare [33]. Moreover, the integration of artificial intelligence (AI) into ECG interpretation holds potential for increasing accuracy and efficiency [28]. AI algorithms can assist in identifying subtle abnormalities that may be overlooked, thereby improving diagnostic precision. Artificial intelligence already plays a significant role in cardiological diagnosis through automated ECG interpretation (available even in some common smartwatches) and automated analysis of echocardiograms (such as in the assessment of myocardial work). The incorporation of genetic testing for inherited cardiac conditions, when combined with appropriate counselling, further strengthens the ability to detect athletes at risk for SCD [34].

Educational initiatives are essential to the successful implementation of any screening program [35]. Raising awareness among athletes, coaches, and healthcare providers about the importance of cardiovascular health and the potential risks associated with intense physical activity is crucial [34]. The COCIS protocols emphasize education as a fundamental component, promoting a culture of proactive health management [4]. By empowering individuals with knowledge, the likelihood of early symptom recognition and timely medical evaluation increases, further enhancing the effectiveness of the screening process [36].

In light of these considerations, the COCIS protocols represent the most professional and medically sound approach to cardiological evaluation in competitive football. They are grounded in robust scientific evidence and have been refined through decades of practical application and research [5]. The protocols balance thorough cardiovascular assessment with minimizing unnecessary interventions [4]. By prioritizing athlete’s safety through early detection and management of cardiac conditions, the COCIS guidelines set a high standard for sports cardiology [37].

The success of the Italian model serves as a compelling example for other nations [16,38]. It may indeed present certain limitations, such as resource constraints in lower-income regions and variability in the clinical expertise required to interpret advanced diagnostics and mitigate the risk of overdiagnosis. However, it demonstrates that with careful implementation, including appropriate resource allocation, education, and support systems, the challenges associated with comprehensive cardiovascular screening can be effectively addressed [23]. The protocols’ emphasis on individualized care ensures that athletes receive recommendations tailored to their specific health profiles, aligning with the best practices in modern medicine [39].

Ultimately, the COCIS protocols embody a commitment to athlete welfare, underpinned by scientific rigor and clinical expertise. They offer a viable and effective strategy for reducing the incidence of sudden cardiac death in competitive sports, affirming their position as the most professional and medicine-driven approach to cardiological fitness assessment in football [23].

## 7. Conclusions

The Italian COCIS protocols for cardiological clearance in competitive football offer a structured model for preventing sudden cardiac death (SCD) in athletes [4]. Through mandatory ECG-based screening and follow-up evaluations, COCIS demonstrates how systematic and proactive prevention can significantly reduce risk, highlighted by an 89% drop in SCD incidence among Italian athletes between 1979–1980 and 2003–2004 [15,40].

As other countries consider similar strategies, the Italian experience underscores the need to balance screening efficacy with the minimization of false positives [28]. Future directions include longitudinal outcome studies, international validation of screening criteria, and the integration of genomic tools for improved risk stratification.

Ultimately, the evolving COCIS framework remains centered on athlete safety, offering a potential foundation for future global standards in sports cardiology [4].

## Figures and Tables

**Table 1:** Common structural and electrical causes of sudden cardiac death (SCD) in athletes.

Condition	Description	Main Risk	Epidemiology	Diagnostic Approach	COCIS Protocol Focus
Hypertro-phic Cardiomyopathy (HCM)	Genetic disorder causing thickening of the left ventricular wall	Arrhythmias and SCD, especially during physical exertion	Most common cause of SCD in young athletes; especially prevalent in Afro-Caribbean athletes	Comprehensive imaging (echocardio-gram, cardiac MRI), regular monitoring	Early screening and distinguishing physiological vs. pathological hypertrophy
Arrhythmogenic Right Ventricular Cardiomyopathy (ARVC)	Genetic condition with progressive replacement of heart muscle by fibrous or fatty tissue, typically affecting the right ventricle	Arrhythmias and SCD, especially between 2nd and 4th decades of life	Prevalence: ~1 in 5000 to 1 in 2000 (Europe)	ECG monitoring, exercise testing, cardiac MRI (especially under stress), advanced imaging when abnormalities or family history present	Early identification of even minor ECG changes; stress imaging when needed
Congenital Coronary Artery Anomalies	Structural abnormalities in coronary arteries present from birth	Ischemia and fatal arrhythmias during exertion	Rare but serious; exact prevalence varies	CT coronary angiography, especially when standard screening is inconclusive	Use of coronary CT angiography when anomalies are suspected
Ion Channelo-pathies (e.g., Long QT Syndrome, Brugada Syndrome)	Inherited disorders affecting the heart’s electrical activity	High-intensity exercise alters the electrical and autonomic balance of the heart, potentially triggering arrythmias in athletes with underlying channelopathies	Rare; varies by specific syndrome	Multidisciplinary approach: clinical assessment, resting ECG, exercise testing, Holter monitoring, pharmacologic stress testing, genetic testing	Routine genetic testing, personalized risk management (training modifications, monitoring, therapy)

**Table 2:** Rare causes of sudden cardiac death (SCD) in athletes.

Condition	Involved Gene	QT Prolongation	Type of Arrhythmias	Risk of SCD	Epidemiology
Andersen–Tawil Syndrome (LQTS type 7)	KCNJ2 (potassium channel, Kir2.1)	Mild to moderate, prominent U waves, possible long QT	Bidirectional VT, polymorphic VT	Present but relatively low compared to other LQTS types	~0.08–0.1 per 100,000 (~1 in 1,000,000)
Timothy Syndrome (LQTS type 8)	CACNA1C (L-type calcium channel)	Severe (QT > 600 ms), often with 2:1 AV block	Torsades de Pointes, polymorphic VT, very high risk of SCD	Extremely high, often fatal in early childhood	~0.0015 per 100,000 (~1–2 in 100 million)

**Table 3:** Comparison of COCIS (Italy), AHA (USA), and ESC (Europe) protocols for athletes.

Aspect	COCIS Protocol (Italy)	AHA Protocol (USA)	ESC Protocol (Europe)
Initial Screening	Mandatory: personal and family history, physical examination, 12-lead resting ECG.	Pre-participation evaluations (PPEs) are commonly required—though their components vary widely by state, school, and league: personal/family history and physical exam are required; ECG is optional.	Recommended: personal/family history, physical examination, and 12-lead resting ECG for all competitive athletes.
Exercise Testing	Required.	Suggested mainly for athletes > 35 years or with cardiovascular risk factors.	Recommended for individuals with symptoms or abnormal screening; especially >35 years old.
Follow-Up	Annual check-ups or more frequent depending on the sport and medical history.	Typically every 2–4 years; more often if symptoms or risk profile changes.	Depends on risk assessment and sporting level; follow-up intervals individualized.
Eligibility Decisions	Strict criteria; disqualification possible in case of high-risk conditions.	Shared decision-making model, considering individual and sport-specific risks.	Risk stratification and tailored eligibility recommendations with patient involvement.

## Data Availability

The original contributions presented in this study are included in the article. Further inquiries can be directed to the corresponding author.

## Abbreviations

The following abbreviations are used in this manuscript:

ARVC	Arrhythmogenic right ventricular cardiomyopathy
COCIS	Commissione di Vigilanza per il controllo dell’Idoneità Sportiva
CT	Computed tomography
DCM	Dilated cardiomyopathy
ECG	Electrocardiogram
HCM	Hypertrophic cardiomyopathy
LGE	Late gadolinium enhancement
MRI	Magnetic resonance imaging
SCD	Sudden cardiac death

## References

[1] Maron B.J., Zipes D.P., Kovacs R.J. (2015). Eligibility and Disqualification Recommendations for Competitive Athletes With Cardiovascular Abnormalities: Preamble, Principles, and General Considerations: A Scientific Statement From the American Heart Association and American College of Cardiology. J. Am. Coll. Cardiol..

[2] Pelliccia A., Zipes D.P., Maron B.J. (2008). Bethesda Conference #36 and the European Society of Cardiology Consensus Recommendations revisited a comparison of U.S. and European criteria for eligibility and disqualification of competitive athletes with cardiovascular abnormalities. J. Am. Coll. Cardiol..

[3] Baggish A.L., Wood M.J. (2011). Athlete’s heart and cardiovascular care of the athlete: Scientific and clinical update. Circulation.

[4] Zeppilli P., Biffi A., Cammarano M., Castelletti S., Cavarretta E., Cecchi F., Colivicchi F., Contursi M., Corrado D., D’aNdrea A. (2024). Italian Cardiological Guidelines (COCIS) for Competitive Sport Eligibility in athletes with heart disease: Update 2024. Minerva Med..

[5] Corrado D., Basso C., Thiene G. (2001). Sudden cardiac death in young people with apparently normal heart. Cardiovasc. Res..

[6] Harmon K.G., Asif I.M., Klossner D., Drezner J.A. (2011). Incidence of sudden cardiac death in National Collegiate Athletic Association athletes. Circulation.

[7] Sheikh N., Sharma S. (2014). Impact of ethnicity on cardiac adaptation to exercise. Nat. Rev. Cardiol..

[8] Corrado D., Basso C., Rizzoli G., Schiavon M., Thiene G. (2003). Does sports activity enhance the risk of sudden death in adolescents and young adults?. J. Am. Coll. Cardiol..

[9] Malhotra A., Sharma S. (2017). Hypertrophic Cardiomyopathy in Athletes. Eur. Cardiol..

[10] Rowin E.J., Maron B.J., Carrick R.T., Patel P.P., Koethe B., Wells S., Maron M.S. (2020). Outcomes in Patients With Hypertrophic Cardiomyopathy and Left Ventricular Systolic Dysfunction. J. Am. Coll. Cardiol..

[11] Marcus F.I., McKenna W.J., Sherrill D., Basso C., Bauce B., Bluemke D.A., Calkins H., Corrado D., Cox M.G., Daubert J.P. (2010). Diagnosis of arrhythmogenic right ventricular cardiomyopathy/dysplasia: Proposed modification of the Task Force Criteria. Eur. Heart J..

[12] Riele A.S.T., James C.A., Philips B., Rastegar N., Bhonsale A., Groeneweg J.A., Murray B., Tichnell C., Judge D.P., Van Der Heijden J.F. (2013). Mutation-positive arrhythmogenic right ventricular dysplasia/cardiomyopathy: The triangle of dysplasia displaced. J. Cardiovasc. Electrophysiol..

[13] D’ANdrea A., Cocchia R., Riegler L., Scarafile R., Salerno G., Gravino R., Golia E., Pezzullo E., Citro R., Limongelli G. (2010). Left ventricular myocardial velocities and deformation indexes in top-level athletes. J. Am. Soc. Echocardiogr..

[14] Lang R.M., Badano L.P., Mor-Avi V., Afilalo J., Armstrong A., Ernande L., Flachskampf F.A., Foster E., Goldstein S.A., Kuznetsova T. (2015). Recommendations for cardiac chamber quantification by echocardiography in adults: An update from the American Society of Echocardiography and the European Association of Cardiovascular Imaging. J. Am. Soc. Echocardiogr..

[15] Corrado D., Basso C., Pavei A., Michieli P., Schiavon M., Thiene G. (2006). Trends in sudden cardiovascular death in young competitive athletes after implementation of a preparticipation screening program. JAMA.

[16] Corrado D., Migliore F., Basso C., Thiene G. (2006). Exercise and the risk of sudden cardiac death. Herz.

[17] Thiene G., Carturan E., Corrado D., Basso C. (2010). Prevention of sudden cardiac death in the young and in athletes: Dream or reality?. Cardiovasc. Pathol..

[18] Baggish A.L., Hutter A.M., Wang F., Yared K., Weiner R.B., Kupperman E., Picard M.H., Wood M.J. (2010). Cardiovascular screening in college athletes with and without electrocardiography: A cross-sectional study. Ann. Intern. Med..

[19] Magnani J.W., Wang N., Nelson K.P., Connelly S., Deo R., Rodondi N., Schelbert E.B., Garcia M.E., Phillips C.L., Shlipak M.G. (2013). Electrocardiographic PR interval and adverse outcomes in older adults: The Health, Aging, and Body Composition study. Circ. Arrhythm. Electrophysiol..

[20] Friedrich M.G., Sechtem U., Schulz-Menger J., Holmvang G., Alakija P., Cooper L.T., White J.A., Abdel-Aty H., Gutberlet M., Prasad S. (2009). Cardiovascular magnetic resonance in myocarditis: A JACC White Paper. J. Am. Coll. Cardiol..

[21] Colbert J.A. (2014). Clinical decisions. Cardiac screening before participation in sports--polling results. N. Engl. J. Med..

[22] Goff N.K., Hutchinson A., Koek W., Kamat D. (2023). Meta-analysis on the Effectiveness of ECG Screening for Conditions Related to Sudden Cardiac Death in Young Athletes. Clin. Pediatr..

[23] Wheeler M.T., Heidenreich P.A., Froelicher V.F., Hlatky M.A., Ashley E.A. (2010). Cost-effectiveness of preparticipation screening for prevention of sudden cardiac death in young athletes. Ann. Intern. Med..

[24] Maron B.J., Friedman R.A., Caplan A. (2015). Ethics of preparticipation cardiovascular screening for athletes. Nat. Rev. Cardiol..

[25] Drezner J., Corrado D. (2011). Is there evidence for recommending electrocardiogram as part of the pre-participation examination?. Clin. J. Sport. Med..

[26] Myerburg R.J., Vetter V.L. (2007). Electrocardiograms should be included in preparticipation screening of athletes. Circulation.

[27] A Drezner J., Ackerman M.J., Anderson J., Ashley E., A Asplund C., Baggish A.L., Börjesson M., Cannon B.C., Corrado D., DiFiori J.P. (2013). Electrocardiographic interpretation in athletes: The ‘Seattle criteria’. Br. J. Sports Med..

[28] Sharma S., Drezner J.A., Baggish A., Papadakis M., Wilson M.G., Prutkin J.M., La Gerche A., Ackerman M.J., Börjesson M., Salerno J.C. (2018). International recommendations for electrocardiographic interpretation in athletes. Eur. Heart J..

[29] Hyde N., Prutkin J.M., Drezner J.A. (2019). Electrocardiogram interpretation in NCAA athletes: Comparison of the ‘Seattle’ and ‘International’ criteria. J. Electrocardiol..

[30] De Mozzi P., Longo U.G., Galanti G., Maffulli N. (2008). Bicuspid aortic valve: A literature review and its impact on sport activity. Br. Med. Bull..

[31] Malhotra A., Dhutia H., Finocchiaro G., Gati S., Beasley I., Clift P., Cowie C., Kenny A., Mayet J., Oxborough D. (2018). Outcomes of Cardiac Screening in Adolescent Soccer Players. N. Engl. J. Med..

[32] Maron B.J., Haas T.S., Murphy C.J., Ahluwalia A., Rutten-Ramos S. (2014). Incidence and causes of sudden death in U.S. college athletes. J. Am. Coll. Cardiol..

[33] Drezner J.A., Sharma S., Baggish A., Papadakis M., Wilson M.G., Prutkin J.M., La Gerche A., Ackerman M.J., Borjesson M., Salerno J.C. (2017). International criteria for electrocardiographic interpretation in athletes: Consensus statement. Br. J. Sports Med..

[34] Casian M., Papadakis M. (2024). Risk Stratification in Sports Cardiology.

[35] Yoon H.J., Kim K.H., Hornsby K., Park J.H., Park H., Kim H.Y., Cho J.Y., Ahn Y., Jeong M.H., Cho J.G. (2021). Gender Difference of Cardiac Remodeling in University Athletes: Results from 2015 Gwangju Summer Universiade. Korean Circ. J..

[36] Corrado D., Pelliccia A., Heidbuchel H., Sharma S., Link M., Basso C., Biffi A., Buja G., Delise P., Gussac I. (2010). Recommendations for interpretation of 12-lead electrocardiogram in the athlete. Eur. Heart J..

[37] Zipes D.P., Link M.S., Ackerman M.J., Kovacs R.J., Myerburg R.J., Estes N.A.M. (2015). Eligibility and Disqualification Recommendations for Competitive Athletes with Cardiovascular Abnormalities: Task Force 9: Arrhythmias and Conduction Defects: A Scientific Statement From the American Heart Association and American College of Cardiology. J. Am. Coll. Cardiol..

[38] Borjesson M., Urhausen A., Kouidi E., Dugmore D., Sharma S., Halle M., Heidbuchel H., Bjornstad H.H., Gielen S., Mezzani A. (2011). Cardiovascular evaluation of middle-aged/ senior individuals engaged in leisure-time sport activities: Position stand from the sections of exercise physiology and sports cardiology of the European Association of Cardiovascular Prevention and Rehabilitation. Eur. J. Cardiovasc. Prev. Rehabil..

[39] Pelliccia A., Fagard R., Bjørnstad H.H., Anastassakis A., Arbustini E., Assanelli D., Biffi A., Borjesson M., Carrè F., Corrado D. (2005). Recommendations for competitive sports participation in athletes with cardiovascular disease: A consensus document from the Study Group of Sports Cardiology of the Working Group of Cardiac Rehabilitation and Exercise Physiology and the Working Group of Myocardial and Pericardial Diseases of the European Society of Cardiology. Eur. Heart J..

[40] Corrado D., Basso C., Schiavon M., Thiene G. (1998). Screening for hypertrophic cardiomyopathy in young athletes. N. Engl. J. Med..

